# The level of moral sensitivity among nurses: a systematic review and meta-analysis

**DOI:** 10.1186/s12912-025-02892-6

**Published:** 2025-03-25

**Authors:** Ting Zhao, Shi Chen, Xiaohui Dong, Xianyin Lu, Xinyu Chen, Hang Li, Shirui Tang, Shasha Wen, Huanle Liu, Chaoming Hou, Jing Gao, Jing Yang

**Affiliations:** College of Nursing, Chengdu University of Traditional Chinese Medicine, Chengdu, 611137 Sichuan China

**Keywords:** Nurses, Moral sensitivity, Education, Meta-analysis

## Abstract

**Background:**

Nurses, the largest frontline healthcare group in the world, experience a high incidence of moral distress. Enhancing moral sensitivity (MS) can effectively alleviate this distress. However, MS levels among nurses have not been clearly defined. Therefore, this study aimed to assess the level of MS among nurses and provide evidence-based insights to improve their moral practices.

**Methods:**

This review searched multiple databases, including PubMed, Cochrane Library, Embase, Web of Science, CINAHL, Scopus, Medline, China Knowledge Resource Integrated Database, Wanfang Database, VIP Database, Chinese Biomedical Database, Chinese Medical Journal Full Text Database, Google Scholar, and OpenGrey, from inception to December 31, 2024. Two reviewers (Ting Zhao and Shi Chen) independently screened the literature and extracted data. Their quality was assessed using the Joanna Briggs Institute’s Critical Appraisal Tool. Data were analyzed using Stata software (version 17.0) to synthesize the mean scores of the moral sensitivity questionnaire (MSQ). Subgroup and meta-regression analysis were performed to identify the sources of heterogeneity, and a sensitivity analysis was conducted to assess the robustness of the results.

**Results:**

A total of 99 studies involving 29,387 nurses were included. The pooled mean score for MS was 4.49 [95% *CI* (4.29, 4.70)], indicating a moderate level. Meta-regression revealed the assessment instrument, country, and department as potential sources of heterogeneity. The mean scores by country were as follows: China (5.22), Korea (4.82), Iran (4.44), and Turkey (3.28). The scores for the different assessment instruments varied, with the MSQ-revised version (5.46) having the highest scores.

**Conclusion:**

Nurses demonstrated moderate MS levels, indicating opportunities for further improvement. This review offers useful insights for nurse managers and educators in shaping strategies to improve moral training.

**Supplementary Information:**

The online version contains supplementary material available at 10.1186/s12912-025-02892-6.

## Introduction

Moral distress was originally defined as the experience of individuals in the workforce knowing or believing in an ethically appropriate course of action yet feeling unable to pursue it because of organizational or other external constraints [1]. As frontline healthcare workers, nurses face heavy workloads and are constrained by the decisions of others in the clinical decision-making process [2]. Compared to other healthcare professionals, they are particularly vulnerable to moral distress [3, 4]. Moral distress has been a significant issue in nursing practice for many decades [5]. Research has demonstrated that moral distress ultimately diminishes nurses’ professional performance, negatively impacting nurses, patients, and healthcare organizations [6]. For nurses, moral distress leads to feelings of guilt and anguish [3], even causing mental health issues [7]. For patients, nurses facing moral distress cannot provide high-quality care, consequently jeopardizing the quality of care, safety, and satisfaction [8–10]. For healthcare organizations, moral distress can increase nurses’ intentions to leave the profession [11–13]. Therefore, measures must be taken to mitigate this negative impact.

Moral sensitivity (MS) has been recognized as both a prerequisite and a foundational component for nurses to make moral decisions and engage in moral behaviors that align with their professional responsibilities [14–16]. Research suggests that enhancing MS is a key strategy for promoting moral behavior and reducing moral distress [9, 12, 17]. According to Lützén et al., MS involves an intuitive understanding of the vulnerable situations of patients by nurses and an awareness of the moral consequences of decisions made on their behalf [18]. Studies have demonstrated that nurses with higher MS levels are more adept at identifying moral issues, making sound moral decisions [19], and exhibiting moral behavior [20]. Consequently, they can not only deliver high-quality, human-centered care to patients, thereby enhancing patient outcomes [21], but also depict greater respect for colleagues, foster a positive work environment, and improve professional identity and job satisfaction [21]. However, nurses with poor MS are less likely to identify moral issues and make appropriate decisions, eventually leading to a vicious cycle of low MS and high moral distress. Previous studies have demonstrated a direct and significant effect of MS on relieving nurses from moral distress [22]. Khaghanizadeh et al. conducted moral decision-making training for nurses, and the results also demonstrated that improving MS through effective training can reduce moral distress in nurses [23–25]. In conclusion, enhancing MS is an effective strategy to reduce moral distress.

To effectively improve MS in nurses, the first step is to accurately assess the MS level and provide targeted measures. Many scholars have devoted themselves to studying the current MS levels among nurses. For instance, Khodaveisi et al. [26] suggested that MS remained at a high level in nurses caring for patients with coronavirus 2019. Huang et al. [27] surveyed 331 nurses and found that the MS among nurses was at a relatively moderate-to-high level. However, Chen et al. [28] discovered that MS among nurses was at a low-to-moderate level. Current studies on MS levels among nurses demonstrate significant discrepancies, which may be related to factors such as department, gender, education level, work experience, and learning experience in MS [29–31]. Although current studies have depicted varying results, it is encouraging that the level of MS among nurses has garnered the attention it deserves. A high MS level not only alleviates moral distress and benefits the professional development of nurses, but also improves the quality of patient care and satisfaction [32]. Consequently, it is important to identify the overall level of MS among nurses to provide evidence for targeted intervention measures. Meta-analysis is a robust method for objectively synthesizing the findings of multiple studies and offers a comprehensive approach to assess the overall effect or trend within a given research domain [33]. However, to date, we have not found any meta-analyses of MS levels among nurses. Consequently, this review aimed to assess the level of MS among nurses, provide evidence-based insights in this area, and facilitate moral practice.

## Methods

The review was registered in PROSPERO (CRD42024573221) and was reported according to the Preferred Reporting Items for Systematic Reviews and Meta-Analysis guidelines [34, 35]. As this was a meta-analysis, ethical approval was not required.

### Search strategy

A systematic search was conducted for studies published in PubMed, Cochrane Library, Embase, Web of Science, CINAHL, Scopus, Medline, China Knowledge Resource Integrated Database, Wanfang Database, VIP Database, Chinese Biomedical Database, Chinese Medical Journal Full Text Database, Google Scholar, and OpenGrey with dates ranging from inception until 31 December 2024. Keywords and subject headings related to MS and nurses were used as search terms, including “nurse*” AND “moral or ethical sensitivity” were used without date restrictions. Simultaneously, the references of all the included studies were browsed and screened to identify additional relevant studies. If the full-text cannot be viewed or downloaded from the electronic databases, we attempt to get the full - text by borrowing resources from other libraries or by contacting the corresponding author. The specific search strategy is presented in Appendix A.

### Inclusion and exclusion criteria

The eligibility criteria were as follows: (1) P (Population): The research subjects were nurses with a certificate of practice qualification; (2) O (Outcome): The MS level among nurses measured by the moral sensitivity questionnaire (MSQ); (3) S (Study design): Cross-sectional study.

The exclusion criteria included the following: (1) Studies with unextractable data on MS of nurses, (2) studies not written in English or Chinese, and (3) studies in which the full text was unavailable.

### Study selection and data extraction

All studies were imported into EndNoteX9 software, duplicate entries were removed, and the title and abstract of each study were screened independently by two reviewers (Ting Zhao and Shi Chen) to identify potential studies. Articles that met the inclusion and exclusion criteria were identified by reading the full text. Two reviewers (Ting Zhao and Shi Chen) cross-checked to determine the final eligible studies, and a third party (Xiaohui Dong) was consulted whenever disagreement arose. For each study, the following information was extracted: (1) study characteristics, including first author, publication year, country, study design, gender, age, department, education level, work experience, and learning experience in MS; (2) meta-analysis data, such as sample size, assessment instrument, and mean and standard deviation.

### Methodological quality assessment

The quality assessment of the studies included in our review was conducted using the Joanna Briggs Institute’s (JBI) Critical Appraisal Tool for cross-sectional studies [36]. This appraisal tool has 8 items that evaluate overall quality from the perspective of sample inclusion criteria, detailed characteristics of the study subject, ways of exposure and outcomes measured, diseases, measuring and dealing with confounding factors, and data analysis. Each item is classified as yes, no, unclear, or not applicable. There are no established guidelines for determining scoring values using the JBI tool [37]. Studies with 6–8 items “yes,” 3–5 items “yes,” and 0–2 items “yes” were ranked as high, moderate, and low quality, respectively [38]. Two reviewers (Ting Zhao and Shi Chen) independently assessed the risk of bias, and any discrepancies were resolved through discussion.

### Data synthesis

Various versions of the MSQ were used in the meta-analysis, each with a different number of items, dimensions, and total scores. Moreover, these various versions used different point Likert scales. To ensure comparability and facilitate meta-analysis, the average mean scores and standard deviations from these assessment instruments were used as the corrected mean scores and standard deviations (referred to as the mean score and standard deviation in this review) [39].Two reviewers (Ting Zhao and Shi Chen) independently performed these transformations, and any discrepancies were resolved through discussion with a third reviewer.

The Stata software (version 17.0) was used to pool the mean scores of standard deviations across studies, and the pooled mean scores were presented with weighted effect sizes and 95% confidence intervals (*CIs*). Heterogeneity among studies was assessed using *I*^2^ statistics, with *I*^*2*^ values of 25%, 50%, and 75% indicating low, moderate, and high heterogeneity, respectively [40]. With *I*^2^ > 50% and *P* < 0.10, indicating significant heterogeneity, a random-effects model was used for analysis; otherwise, a fixed-effects model was used. If heterogeneity existed, meta-regression and subgroup analyses were performed. Based on previous evidence, we considered the following study characteristics as potential sources of heterogeneity: Country, department, assessment instrument, gender, education level, work experience, learning experience in MS, publication year, and sample size. As meta-regression was performed only for covariates reported in at least ten studies [40], the following variables were tested in meta-regression: Publication year, sample size, assessment instrument, country, and department. A sensitivity analysis was conducted by sequentially excluding individual studies to evaluate the robustness of the results. Egger’s tests were utilized to examine potential publication bias, with *P* > 0.05 indicating a low likelihood of publication bias [41]. When publication bias emerged, the “cut-and-fill method” was used.

## Results

### Study search and selection results

**Fig. 1:**
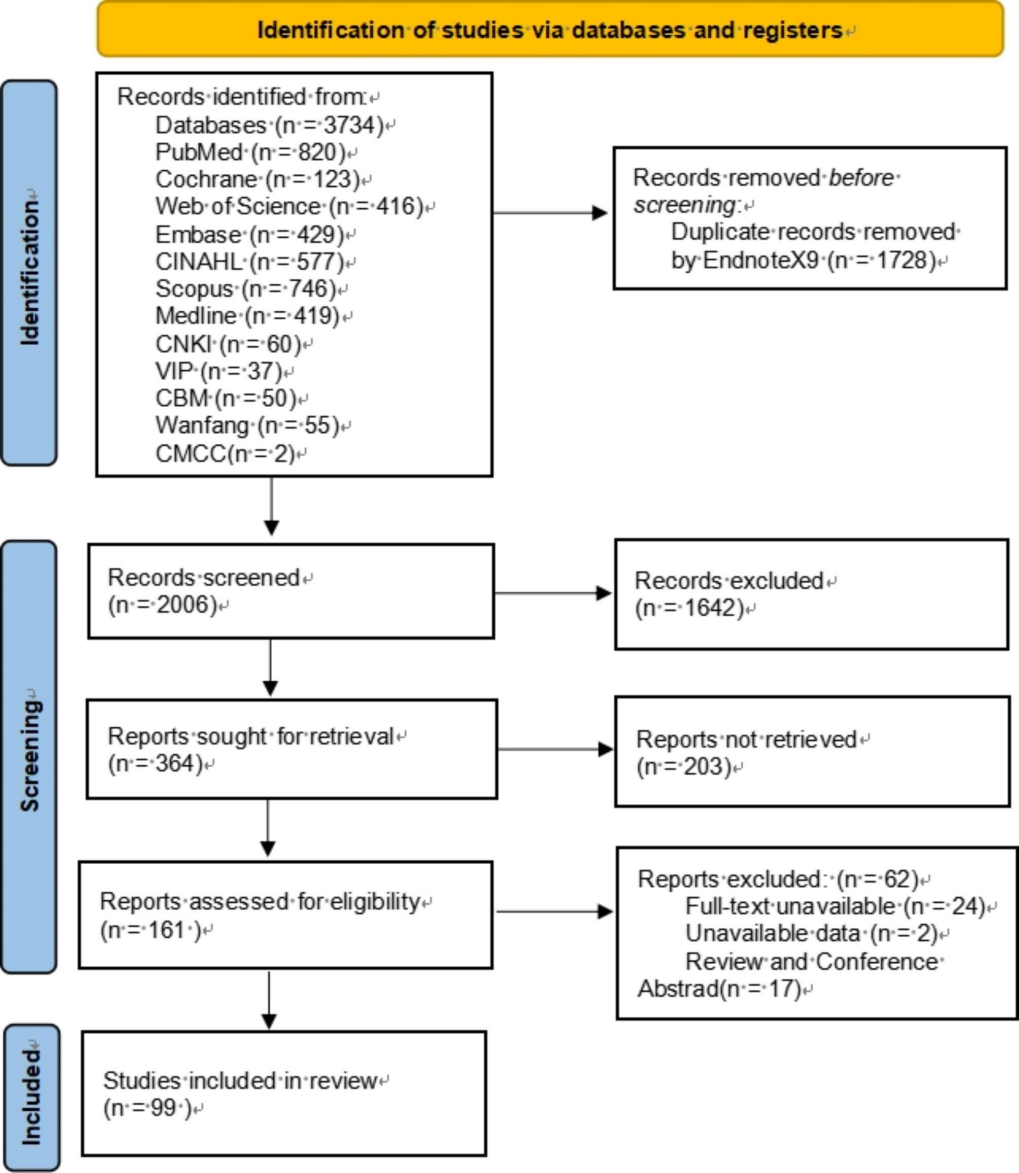
Literature screening process diagram

A total of 3,734 records were retrieved from the electronic database. No new literature were identified in the reference lists of the included studies. The titles and abstracts of the remaining 2,006 studies were screened after deduplication using the EndNoteX9 software. We then read the full texts of 161 potentially relevant studies, and 99 studies [13, 20, 24–27, 29, 31, 42–132] ultimately met the inclusion criteria. A screening flowchart is depicted in Fig. 1.

### Summary of included studies

A total of 99 studies [13, 20, 24–27, 29, 31, 42–132], were conducted across 5 different countries: Iran (*n* = 28) [24–26, 44, 47, 51, 58, 62, 66, 68, 69, 74, 82–92, 95, 110, 111, 113, 115], Turkey (*n* = 24) [42, 54, 57, 61, 65, 70, 71, 75, 76, 78–81, 100–107, 109, 110, 132], China (*n* = 35) [20, 27, 30, 43, 45, 46, 48–50, 52, 53, 55, 56, 59, 72, 96–99, 116–131], Korea (*n* = 11) [13, 31, 60, 63, 64, 73, 77, 93, 94, 112, 114], and Spain (*n* = 1) [67]. The review included 29,387 nurses with sample sizes ranging from from 49 to 1,094. The studies were published between 2012 and 2024, and all were cross-sectional. Most studies were conducted in general hospitals without distinguishing between specific nursing departments, although some scholars focused on ICU nurses, and few studies examined the MS levels of nurses in hospice, pediatric, and psychiatric settings.

This review included several versions of the MSQ, such as the original MSQ, MSQ-Revised (MSQ-R) version, Lützén MSQ (L-MSQ), the Korean version of the MSQ (K-MSQ), and Chinese MSQ-Revised version (MSQR-CV). These assessment instruments utilize various Likert scales, including 7-, 6-, 5-point, and mixed 7- and 6-point scales.

To ensure consistency, data from 60 studies [20, 24, 25, 27, 29, 30, 43, 45–50, 52, 53, 55, 56, 58, 59, 62, 66–68, 70, 72, 82–92, 96–98, 104, 110, 111, 113, 115–131] that did not use a 7-point Likert scale were transformed. Additionally, 78 studies [24–27, 29, 42–59, 61–63, 66–68, 70–72, 74–76, 78, 80–92, 95–103, 105, 107–110, 113, 115–124, 127, 128, 131, 132] reported only the total mean scores for MS, which were also transformed. A summary of the detailed characteristics of the included studies is presented in Table 1.

**Table 1:** Main characteristics of all eligible studies

Author (year)	Country	Study design	Sample size (female/male)	Age (years)	Department	Education level (*n*)	Work experience (*n*)	Learning experiences in MS (*n*)	Instrument (Likert/entries)	Average mean score (total mean score)
Ahansaz et al. [44] (2024)	Iran	cross-sectional study	202(88/114)	30.95 ± 6.03	Mixed	NR	NR	NR	MSQ (7/30)	4.97 ± 0.72 (149.07 ± 21.60)
Tiryaki et al. [42] (2024)	Turkey	cross-sectional study	398(287/111)	24.24 ± 2.97	Mixed	Bachelor or lower (398)	≤ 5year (287)	NR	MSQ (7/30)	3.02 ± 0.86 (90.49 ± 25.94)
Yildirim et al. [54] (2022)	Turkey	cross-sectional study	245(209/36)	NR	Mixed	NR	NR	NR	MSQ (7/30)	3.10 ± 0.75 (92.89 ± 22.49)
Darzi-Ramandi et al. [51] (2023)	Iran	cross-sectional study	211(166/45)	NR	Mixed	Bachelor or lower (182) Master or above (29)	NR	NR	MSQ (7/30)	3.72 ± 0.54 (111.61 ± 16.10)
Üzar Özçetin et al. [57] (2022)	Turkey	cross-sectional study	120(106/14)	NR	Mixed	NR	NR	NR	MSQ (7/30)	6.13 ± 0.72 (183.86 ± 21.52)
Taylan et al. [61] (2021)	Turkey	cross-sectional study	156(144/12)	NR	ICU	NR	NR	NR	MSQ (7/30)	2.78 ± 0.61 (83.37 ± 18.31)
Cerit et al. [65] (2021)	Turkey	cross-sectional study	226(192/34)	31.03 ± 7.47	Mixed	NR	NR	NR	MSQ (7/30)	3.41 ± 0.89 (NR)
Kavurmacı et al. [71] (2019)	Turkey	cross-sectional study	102(75/27)	27.68 ± 4.86	ICU	Bachelor or lower (102)	NR	Yes (73) No (29)	MSQ (7/30)	2.74 ± 0.70 (82.08 ± 21.13)
Palazoglu et al. [70] (2019)	Turkey	cross-sectional study	236(112/124)	NR	Emergency	Bachelor or lower (224) Master or above (12)	NR	NR	MSQ (7/30)	3.01 ± 0.60 (90.40 ± 18.10)
Basar et al. [75] (2019)	Turkey	cross-sectional study	160(132/28)	30.10 ± 6.20	ICU	Bachelor or lower (152) Master or above (8)	NR	NR	MSQ (7/30)	3.03 ± 0.66 (90.90 ± 19.90)
Goktas et al. [76] (2023)	Turkey	cross-sectional study	362(296/66)	21.60 ± 4.24	ICU	Bachelor or lower (312) Master or above (50)	≤ 5year (50) ≥ 10years (62)	NR	MSQ (7/30)	3.02 ± 0.96 (90.70 ± 28.89)
Goo et al. [77] (2024)	Korea	cross-sectional study	167(161/6)	31.16 ± 5.79	Pediatric	NR	NR	NR	MSQ (7/30)	5.02 ± 0.59 (NR)
Arslan et al. [78] (2018)	Turkey	cross-sectional study	200(178/22)	NR	Pediatric	Bachelor or lower (81)	≤ 5year (93) ≥ 10years (66)	NR	MSQ (7/30)	3.20 ± 0.81 (95.89 ± 24.34)
Kumsar et al. [79] (2021)	Turkey	cross-sectional study	689(606/83)	NR	Mixed	Bachelor or lower (611) Master or above (78)	≥ 10years (357)	Yes (431) No (95)	MSQ (7/30)	3.08 ± 0.78 (NR)
Kulakaç et al. [80] (2023)	Turkey	cross-sectional study	268(220/48)	28.08 ± 5.34	Mixed	NR	NR	NR	MSQ (7/30)	3.51 ± 0.63 (105.20 ± 18.80)
Erden Melikoğlu et al. [81] (2023)	Turkey	cross-sectional study	175(150/25)	28.89 ± 7.73	ICU	NR	NR	NR	MSQ (7/30)	2.65 ± 0.66 (79.52 ± 19.76)
Tang et al. [100] (2023)	China	cross-sectional study	331(254/77)	33.34 ± 7.52	Psychiatry	NR	NR	NR	MSQ (7/30)	3.80 ± 0.60 (114.03 ± 18.01)
Sahiner. [109] (2024)	Turkey	cross-sectional study	350(229/121)	32.96 ± 7.41	Mixed	Bachelor or lower (133)	≥ 10years (165)	NR	MSQ (7/30)	2.95 ± 0.88 (88.36 ± 26.33)
Kaya et al. [106] (2022)	Turkey	cross-sectional study	171(141/30)	29.71 ± 7.36	Mixed	NR	NR	NR	MSQ (7/30)	2.58 ± 0.63 (77.5 ± 18.8)
Durmaz et al. [102] (2023)	Turkey	cross-sectional study	131(94/37)	35.36 ± 6.37	Psychiatry	NR	NR	NR	MSQ (7/30)	2.87 ± 0.67 (86.01 ± 20.01)
Kovanci et al. [107] (2024)	Turkey	cross-sectional study	302(274/28)	NR	Mixed	NR	NR	NR	MSQ (7/30)	2.94 ± 0.76 (NR)
Ozdemir et al. [108] (2019)	Turkey	cross-sectional study	351(NR/NR)	NR	Mixed	NR	NR	NR	MSQ (7/30)	2.61 ± 0.59 (78.23 ± 17.66)
Shahvali et al. [111] (2018)	Iran	cross-sectional study	77(NR/NR)	NR	ICU	NR	NR	NR	MSQ (5/30)	4.43 ± 0.56 (88.00 ± 10.51)
Kandemir et al. [104] (2024)	Turkey	cross-sectional study	201(147/54)	NR	Mixed	NR	NR	NR	MSQ (7/30)	2.98 ± 0.64 (89.33 ± 19.33)
Ilter et al. [103] (2024)	Turkey	cross-sectional study	144(95/49)	NR	ICU	Bachelor or lower (144)	≥ 10years (30)	NR	MSQ (7/30)	4.16 ± 0.24 (124.88 ± 7.08)
Cerit et al. [101] (2019)	Turkey	cross-sectional study	99(78/21)	NR	Mixed	NR	NR	NR	MSQ (7/30)	5.00 ± 0.85 (150.05 ± 25.41)
Sevinç et al. [110] (2024)	Turkey	cross-sectional study	713(581/132)	NR	Mixed	NR	NR	NR	MSQ (7/30)	2.56 ± 0.90 (76.76 ± 26.91)
Karatepe et al. [105] (2022)	Turkey	cross-sectional study	384(317/67)	NR	Mixed	NR	NR	NR	MSQ (5/30)	5.19 ± 0.40 (3.79 ± 0.60)
Rezapour-Mirsaleh et al. [58] (2022)	Iran	cross-sectional study	162(103/59)	33.70 ± 6.47	Mixed	NR	NR	NR	MSQ-R (6/9)	5.46 ± 1.85 (41.12 ± 14.05)
Suazo et al. [67] (2020)	Spanish	cross-sectional study	330(277/53)	32.30 ± 7.54	Mixed	NR	NR	NR	MSQ-R (6/9)	5.58 ± 0.93 (NR)
Tang et al. [117] (2024)	China	cross-sectional study	293(233/60)	NR	Mixed	NR	NR	NR	MSQ-R (6/9)	5.32 ± 0.74 (40.01 ± 5.70)
Nobahar et al. [47] (2023)	Iran	cross-sectional study	200(158/42)	32.70 ± 5.65	ICU	NR	NR	NR	L-MSQ (5/25)	3.89 ± 0.54 (64.19 ± 13.43)
Sepehrirad et al. [62] (2021)	Iran	cross-sectional study	132(92/40)	33.99 ± 5.85	Operating room	NR	NR	NR	L-MSQ (5/25)	4.92 ± 0.81 (81.41 ± 20.22)
Afrasiabifar et al. [66] (2021)	Iran	cross-sectional study	250(195/55)	32.60 ± 4.90	Mixed	NR	NR	NR	L-MSQ (5/25)	3.61 ± 0.44 (59.50 ± 11.10)
Khalighi et al. [68] (2020)	Iran	cross-sectional study	110(63/47)	NR	ICU	Bachelor or lower (61) Master or above (49)	NR	NR	L-MSQ (5/25)	3.59 ± 0.51 (59.21 ± 12.65)
Lotfi-Bejestani et al. [24] (2023)	Iran	cross-sectional study	500(377/112)	37.59 ± 7.03	Psychiatry	NR	NR	NR	L-MSQ (5/25)	3.63 ± 0.87 (59.81 ± 13.77)
Rahnama et al. [82] (2017)	Iran	cross-sectional study	204(182/22)	34.00 ± 3.80	Mixed	NR	NR	NR	L-MSQ (5/25)	4.19 ± 0.38 (69.15 ± 5.70)
Borhani et al. [83] (2015)	Iran	cross-sectional study	153(118/35)	32.90 ± 6.52	ICU	NR	NR	NR	L-MSQ (5/25)	4.16 ± 0.51 (68.60 ± 7.80)
Mohammadi et al. [84] (2022)	Iran	cross-sectional study	524(209/315)	33.89 ± 6.91	Mixed	Bachelor or lower (507) Master or above (17)	NR	NR	L-MSQ (5/25)	5.64 ± 0.20 (93.41 ± 2.68)
Hajibabaee et al. [85] (2022)	Iran	cross-sectional study	406(339/67)	37.66 ± 8.56	Mixed	NR	NR	NR	L-MSQ (5/25)	3.76 ± 0.46 (62.06 ± 11.49)
Nazari et al. [86] (2022)	Iran	cross-sectional study	445(225/220)	39.41 ± 9.61	Mixed	Bachelor or lower (258) Master or above (187)	≤ 5year (373)	NR	L-MSQ (5/25)	3.18 ± 0.66 (52.29 ± 16.44)
Vasli et al. [87] (2024)	Iran	cross-sectional study	145(116/29)	35.14 ± 8.36	Mixed	NR	NR	NR	L-MSQ (5/25)	3.85 ± 0.97 (63.45 ± 15.48)
Fouladi et al. [88] (2024)	Iran	cross-sectional study	345(287/58)	32.19 ± 3.98	Mixed	NR	NR	NR	L-MSQ (5/25)	4.29 ± 0.69 (70.75 ± 10.83)
Zahednezhad et al. [89] (2021)	Iran	cross-sectional study	181(138/43)	30.90 ± 4.90	ICU	NR	NR	NR	L-MSQ (5/25)	4.29 ± 0.50 (70.85 ± 7.73)
Khorany et al. [90] (2024)	Iran	cross-sectional study	250(139/111)	NR	Mixed	NR	NR	NR	L-MSQ (5/25)	3.87 ± 0.58 (63.85 ± 8.92)
Sedghi Goyaghaj et al. [91] (2022)	Iran	cross-sectional study	160(99/61)	34.96 ± 7.83	Mixed	NR	NR	NR	L-MSQ (5/25)	4.94 ± 0.13 (81.71 ± 18.19)
Sharifnia et al. [92] (2024)	Iran	cross-sectional study	330(273/57)	35.65 ± 6.53	Mixed	NR	≤ 5 years (66) 6-10years (98) ≥ 10years (166)	NR	L-MSQ (5/25)	4.44 ± 0.63 (73.37 ± 9.78)
Bordbar et al. [114] (2024)	Iran	cross-sectional study	385(256/129)	30.41 ± 8.15	Mixed	NR	≥ 10years (385)	NR	L-MSQ (5/25)	4.33 ± 0.42 (71.55 ± 6.39)
Beiranvanda et al. [25] (2024)	Iran	cross-sectional study	210(NR/NR)	36.01 ± 7.84	Oncology	Bachelor or lower (21) Master or above (189)	NR	NR	L-MSQ (5/25)	3.54 ± 0.84 (58.40 ± 13.30)
Alamdar et al. [112] (2024)	Iran	cross-sectional study	71(NR/NR)	33.98 ± 4.91	ICU	NR	NR	NR	L-MSQ (5/25)	4.83 ± 1.44 (NR)
			109(NR/NR)		Emergency					4.89 ± 1.72 (NR)
Moayedi et al. [116] (2022)	Iran	cross-sectional study	221(204/17)	NR	Mixed	Bachelor or lower (213) Master or above (8)	≥ 10years (77)	NR	L-MSQ (5/25)	5.89 ± 2.72 (67.44 ± 3.52)
Kim et al. [13] (2023)	Korea	cross-sectional study	123(119/4)	35.45 ± 7.60	Hemodialysis	NR	NR	NR	K-MSQ (7/27)	4.88 ± 0.57 (NR)
Kim et al. [60] (2022)	Korea	cross-sectional study	220(208/12)	NR	Mixed	NR	NR	NR	K-MSQ (7/27)	4.82 ± 0.19 (130.17 ± 4.82)
Lim et al. [63] (2021)	Korea	cross-sectional study	171(162/9)	33.30 ± 8.10	Mixed	Bachelor or lower (151) Master or above (20)	≤ 5 years (60) 6-10years (52) ≥ 10years (59)	Yes (143) No (27)	K-MSQ (7/27)	4.80 ± 0.50 (125.3 ± 13.5)
Jeong et al. [64] (2021)	Korea	cross-sectional study	120(111/9)	33.88 ± 9.41	Mixed	NR	NR	NR	K-MSQ (7/27)	4.92 ± 0.49 (NR)
Kim et al. [73] (2013)	Korea	cross-sectional study	303(NR)	30.35 ± 6.95	Mixed	NR	NR	NR	K-MSQ (7/27)	5.14 ± 0.55 (NR)
Kim et al. [93] (2017)	Korea	cross-sectional study	163(137/26)	29.60 ± 6.99	Mixed	NR	NR	NR	K-MSQ (7/27)	5.00 ± 0.61 (NR)
Ahn et al. [94] (2022)	Korea	cross-sectional study	166(152/14)	36.46 ± 9.30	Psychiatry	NR	NR	NR	K-MSQ (7/27)	5.16 ± 0.41 (NR)
Bong et al. [95] (2024)	Korea	cross-sectional study	209(201/8)	NR	Mixed	NR	NR	NR	K-MSQ (7/27)	4.91 ± 0.53 (NR)
Bae et al. [113] (2024)	Korea	cross-sectional study	49(NR/NR)	34.8 ± 10.04	Mixed	NR	NR	NR	K-MSQ (7/27)	5.12 ± 0.54 (NR)
			54(NR/NR)		ICU					5.13 ± 0.64 (NR)
			99(NR/NR)		Geriatrics					5.24 ± 0.54 (NR)
Jo et al. [115] (2015)	Korea	cross-sectional study	344(NR/NR)	29.71 ± 7.39	Mixed	NR	NR	NR	MSQ (7/28)	3.49 ± 0.37 (NR)
Khodaveisi et al. [26] (2021)	Iran	cross-sectional study	405(220/185)	NR	Mixed	NR	NR	NR	MSQ (7/28)	6.38 ± 0.07 (178.61 ± 1.98)
Amiri et al. [69] (2020)	Iran	cross-sectional study	198(191/7)	31.19 ± 5.89	Mixed	NR	NR	NR	MSQ (7/28)	4.62 ± 0.78 (NR)
Momennasab et al. [96] (2023)	Iran	cross-sectional study	298(227/71)	30.69 ± 5.74	ICU	NR	NR	NR	MSQ (7/28)	4.79 ± 0.32 (134.08 ± 9.05)
Jaafarpour et al. [74] (2012)	Iran	cross-sectional study	120(80/40)	34.00 ± 5.20	Mixed	NR	NR	NR	MSQ (7/27)	3.08 ± 0.22 (112.3 ± 11.20)
Karaca et al. [133] (2024)	Turkey	cross-sectional study	483(281/202)	31.67 ± 7.22	Mixed	NR	≤ 5 years (170) 6-10years (125) ≥ 10years (188)	NR	MSQ (7/27)	4.16 ± 0.41 (83.05 ± 6.02)
Zhang et al. [20] (2020)	China	cross-sectional study	525(505/20)	NR	Mixed	NR	NR	NR	MSQ-R (5/9)	5.46 ± 0.15 (NR)
Huang^a^ et al. [27] (2024)	China	cross-sectional study	341(334/7)	NR	Mixed	NR	NR	NR	MSQR-CV (6/9)	5.66 ± 0.84 (42.61 ± 6.46)
Huang^b^ et al. [43] (2024)	China	cross-sectional study	197(158/39)	NR	Neurosurgery	Bachelor or lower (173) Master or above (24)	≤ 5 years (84) 6-10years (69) ≥ 10years (44)	Yes (197) No (197)	MSQR-CV (6/9)	4.62 ± 0.84 (35.20 ± 7.57)
Wu et al. [45] (2023)	China	cross-sectional study	247(201/46)	NR	Mixed	NR	NR	NR	MSQR-CV (6/9)	4.81 ± 0.38 (36.27 ± 3.02)
Pan et al. [46] (2023)	China	cross-sectional study	432(365/67)	NR	Operating room	NR	NR	NR	MSQR-CV (6/9)	5.34 ± 0.68 (40.24 ± 5.28)
Liu et al. [48] (2023)	China	cross-sectional study	230(NR)	NR	Hospice	NR	NR	NR	MSQR-CV (6/9)	4.75 ± 0.82 (35.78 ± 6.34)
Li et al. [49] (2023)	China	cross-sectional study	438(417/21)	NR	Pediatric	NR	NR	Yes (333) No (105)	MSQR-CV (6/9)	4.85 ± 0.87 (36.51 ± 6.71)
He et al. [50] (2023)	China	cross-sectional study	450(NR)	NR	Psychiatry	NR	≤ 5 years (98) 6-10years (115) ≥ 10years (237)	Yes (401) No (49)	MSQR-CV (6/9)	5.22 ± 0.78 (39.29 ± 6.05)
Cheng et al. [52] (2023)	China	cross-sectional study	246(NR)	NR	ICU	NR	NR	NR	MSQR-CV (6/9)	4.78 ± 1.19 (35.98 ± 9.07)
Zhou [53] (2022)	China	cross-sectional study	485(452/33)	NR	Pediatric	Bachelor or lower (190)	≤ 5 years (250) 6-10years (124) ≥ 10years (111)	NR	MSQR-CV (6/9)	5.09 ± 1.04 (38.34 ± 7.95)
Ye et al. [55] (2022)	China	cross-sectional study	404(371/33)	NR	ICU	Bachelor or lower (391) Master or above (13)	≤ 5 years (72) ≥ 10years (54)	Yes (137) No (267)	MSQR-CV (6/9)	5.23 ± 0.94 (39.41 ± 7.21)
Wu et al. [56] (2022)	China	cross-sectional study	184(171/13)	NR	Hospice	Bachelor or lower (179) Master or above (5)	≤ 5 years (150)	NR	MSQR-CV (6/9)	4.94 ± 0.71 (37.21 ± 5.51)
Ouyang et al. [59] (2022)	China	cross-sectional study	305(273/32)	NR	Mixed	Bachelor or lower (140)	≥ 10years (155)	Yes (119) No (65)	MSQR-CV (6/9)	5.52 ± 1.08 (41.59 ± 8.25)
Chen et al. [29] (2022)	China	cross-sectional study	422(415/7)	29.86 ± 5.99	Mixed	NR	NR	NR	MSQR-CV (6/9)	4.75 ± 1.07 (35.82 ± 8.17)
Huang^c^ et al. [72] (2016)	China	cross-sectional study	306(295/11)	29.16 ± 6.27	Mixed	NR	NR	NR	MSQR-CV (6/9)	5.34 ± 0.92 (40.22 ± 7.08)
Zheng et al. [97] (2024)	China	cross-sectional study	349(344/5)	NR	Oncology	NR	NR	NR	MSQR-CV (6/9)	6.03 ± 0.84 (45.36 ± 6.43)
Guo et al. [98] (2024)	China	cross-sectional study	212(147/65)	35.35 ± 7.51	Mixed	NR	NR	NR	MSQR-CV (6/9)	6.19 ± 0.98 (46.62 ± 7.55)
Jiang et al. [99] (2021)	China	cross-sectional study	399(337/62)	31.53 ± 6.26	Mixed	NR	NR	NR	MSQR-CV (6/9)	5.98 ± 0.92 (45.00 ± 7.09)
Jia et al. [119] (2024)	China	cross-sectional study	182(133/49)	NR	ICU	NR	NR	NR	MSQR-CV (6/9)	5.96 ± 0.84 (44.89 ± 6.43)
Huang^d^ et al. [122] (2024)	China	cross-sectional study	524(521/3)	NR	Oncology	Bachelor or lower (519) Master or above (5)	6-10years (130) ≥ 10years (163)	Yes (462) No (62)	MSQR-CV (6/9)	5.92 ± 1.00 (44.57 ± 7.63)
Zhang et al. [124] (2024)	China	cross-sectional study	274(226/48)	NR	ICU	NR	NR	NR	MSQR-CV (6/9)	5.45 ± 1.06 (41.01 ± 8.08)
Zhang et al. [123] (2016)	China	cross-sectional study	476(456/20)	NR	Mixed	NR	NR	NR	MSQR-CV (6/9)	4.58 ± 0.29 (NR)
Wang et al. [125] (2022)	China	cross-sectional study	1094(1052/42)	NR	Mixed	NR	NR	NR	MSQR-CV (6/9)	4.62 ± 0.98 (34.82 ± 7.49)
Meng [130] (2023)	China	cross-sectional study	348(288/60)	NR	Mixed	NR	NR	NR	MSQR-CV (6/9)	3.44 ± 0.77 (NR)
Peng [131] (2023)	China	cross-sectional study	367(352/15)	34.44 ± 6.79	Mixed	Bachelor or lower (347) Master or above (20)	NR	NR	MSQR-CV (6/9)	5.19 ± 0.64 (NR)
Bai et al. [127] (2023)	China	cross-sectional study	516(501/15)	NR	Mixed	Bachelor or lower (510) Master or above (6)	≥ 10years (310)	NR	MSQR-CV (6/9)	5.32 ± 0.36 (NR)
Bai [126] (2023)	China	cross-sectional study	642(622/20)	NR	Mixed	Bachelor or lower (635) Master or above (7)	≥ 10years (186)	NR	MSQR-CV (6/9)	5.28 ± 0.36 (NR)
Liu [129] (2023)	China	cross-sectional study	193(184/9)	NR	Oncology	NR	NR	NR	MSQR-CV (6/9)	4.82 ± 0.74 (36.30 ± 5.75)
Dong et al. [128] (2024)	China	cross-sectional study	471(462/9)	NR	Mixed	NR	NR	NR	MSQR-CV (6/9)	5.70 ± 1.05 (42.92 ± 8.05)
Qi et al. [132] (2024)	China	cross-sectional study	302(270/32)	NR	Mixed	NR	NR	NR	MSQR-CV (6/9)	6.21 ± 0.74 (46.74 ± 5.69)
Xu et al. [121] (2024)	China	cross-sectional study	228(181/47)	23.78 ± 2.07	Mixed	NR	NR	NR	MSQR-CV (6/9)	5.41 ± 0.03 (40.71 ± 0.39)
Pang et al. [120] (2024)	China	cross-sectional study	321(277/44)	30.77 ± 5.33	ICU	Bachelor or lower (67)	NR	Yes (198) No (123)	MSQR-CV (6/9)	5.72 ± 0.77 (43.04 ± 5.95)
Chen et al. [118] (2024)	China	cross-sectional study	465(442/23)	31.90 ± 6.43	Mixed	NR	NR	NR	MSQR-CV (6/9)	5.27 ± 0.84 (39.69 ± 6.50)

Note: SD, standard deviation; NR, not reported

### Quality assessment

The methodological quality of the studies included ranged from moderate to high. All studies clearly defined their subjects, inclusion criteria, measurement methods, statistical analyses, and outcomes. However, 61 studies [13, 20, 24, 26, 27, 29, 31, 44–48, 52, 54, 57, 58, 60–62, 64–67, 69, 72–74, 77, 80–83, 85, 87–91, 93–98, 100, 101, 103, 104, 106, 109, 110, 112, 114, 120, 122–124, 127–129, 131] did not account for the confounding factors in measurement and analysis. Based on the quality assessment, 38 studies [25, 42, 43, 49–51, 53, 55, 56, 59, 63, 68, 70, 71, 75, 76, 78, 79, 84, 86, 92, 99, 102, 105, 107, 108, 111, 113, 115–119, 121, 125, 126, 130, 132] were categorized as high-quality, while 61 studies [13, 20, 24, 26, 27, 29, 31, 44–48, 52, 54, 57, 58, 60–62, 64–67, 69, 72–74, 77, 80–83, 85, 87–91, 93–98, 100, 101, 103, 104, 106, 109, 110, 112, 114, 120, 122–124, 127–129, 131] were categorized as moderate quality. The results of the quality assessment are presented in Appendix B.

### Meta-analysis of MS

**Fig. 2:**
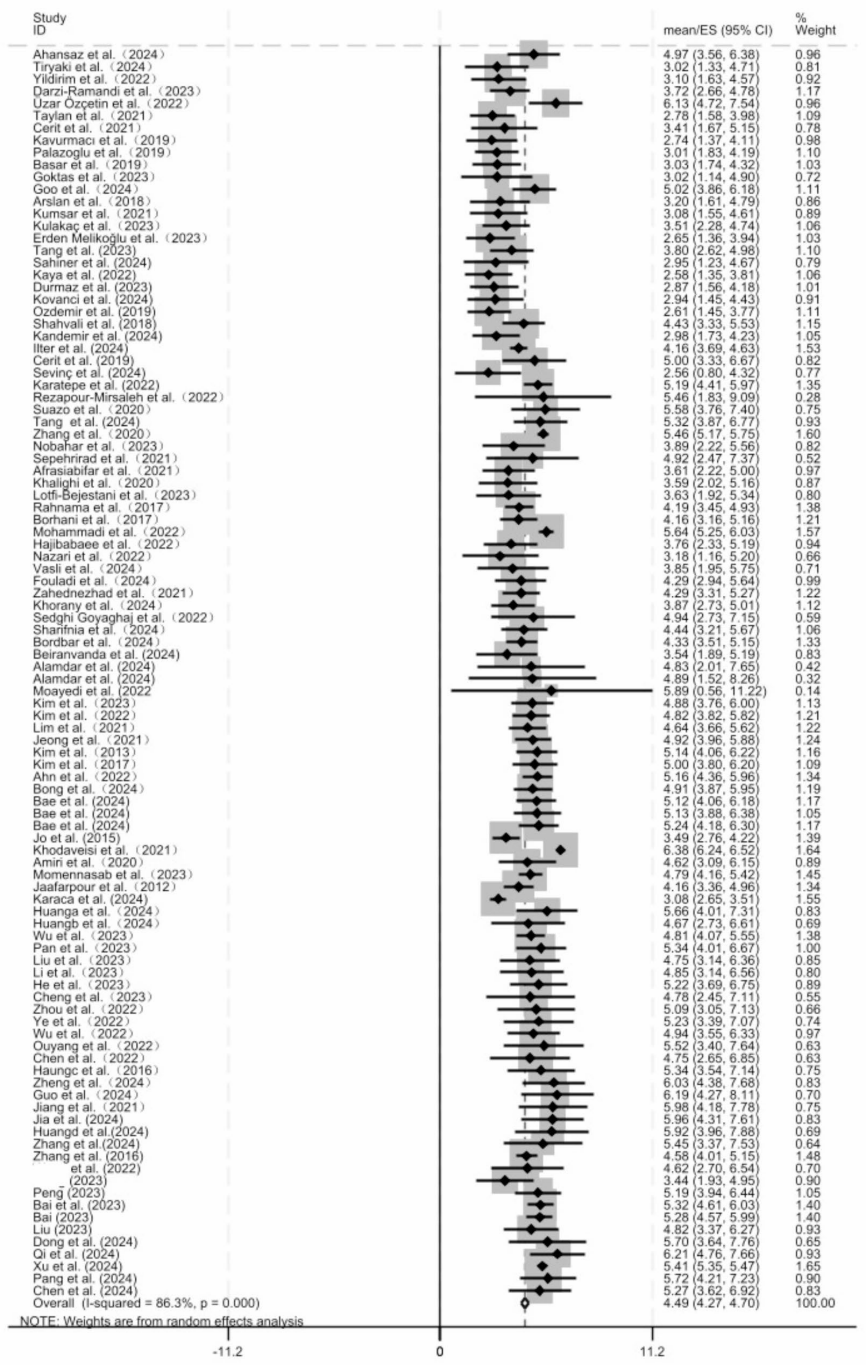
Forest plot of pooled mean total scores for moral sensitivity

A total of 99 studies [13, 20, 24–27, 29, 31, 42–132] were included in the meta-analysis. The *I*^2^ statistic was 86.3%, indicating high heterogeneity. Using a random-effects model, the pooled mean MS score among nurses was 4.49 [95% *CI* (4.29, 4.70)], suggesting a moderate MS level. The results are depicted in Fig. 2.

### Meta-regression analysis results

A meta-regression analysis was conducted to further explore the sources of heterogeneity. The results indicated that the publication year (*P* = 0.23), sample size (*P* = 0.36), assessment instrument (*P* = 0.00), country (*P* = 0.00), and department (*P* = 0.00) were significant factors. Specifically, the assessment instrument, country, and department were identified as potential sources of heterogeneity (Table 2).

**Table 2:** Meta-regression analysis of the level of Nurse’s moral sensitivity

Covariates	β (95%CI)	SE	Z	*P*-value
Publication year	0.44	0.38	1.15	0.23
Sample size	0.001	0.001	0.92	0.36
Assessment instrument	5.55	0.36	15.54	0.00
Country	5.58	1.08	5.16	0.00
Department	4.67	1.27	3.68	0.00

### Subgroup analysis results

A subgroup analysis was conducted based on country, assessment instrument, department, gender, education level, work experience, and learning experience in MS.

Significant differences were found in the subgroups of country and assessment instrument (*P*^*a*^ < 0.01). There was a significant difference in the MS levels among nurses from different countries (*P*^*a*^ < 0.01). China exhibited the highest pooled mean score (China), followed by Korea (4.82), Iran (4.44), and Turkey (3.28). Regarding the assessment instrument used, significant heterogeneity was found (*P*^*a*^ < 0.01). The pooled mean scores ranked from high to low were MSQ-R (5.46), MSQR-CV (5.22), K-MSQ (4.81), L-MSQ (4.23), and MSQ (3.57).

The subgroups of department, gender, educational level, work experience, and learning experience in MS were found to be statistically non-significant (*P*^*a*^ > 0.01). Notably, *I²* statistics indicated substantial variability, with values of 35.76% and 0.00%, respectively, depicting differences between nurses with and without learning experience in MS. The results of subgroup analyses are presented in Table 3.

**Table 3:** Subgroup analysis of the level of Nurse’s moral sensitivity

Subgroups	Number of studies (*N*)	Sample size (*n*)	Effect mode	Pooled mean score (95%CI)	*P* ^a^	Heterogeneity			
						I^2^ (%)	Q		*P* ^b^
Country					0.00				
China	35	13,199	Random	5.22(5.02,5.42)		22.99		32.43	0.00
Iran	28	7,004	Random	4.44(4.09,4.80)		72.14		245.45	0.00
Turkey	24	6,666	Random	3.28(2.98,3.75)		61.34		60.17	0.00
Korea	11	2,188	Random	4.82(4.48,5.16)		32.13		15.42	0.00
Spanish	1	330	Random	5.58(3.76,7.40)		-		-	0.00
Assessment instrument					0.00				
K-MSQ	12	2,021	Random	4.81(4.45,5.17)		34.93		15.24	0.00
L-MSQ	21	5,331	Random	4.23(3.79,4.68)		57.63		42.50	0.00
MSQ	28	7,171	Random	3.57(3.20,3.94)		59.15		65.66	0.00
MSQ-27item	2	603	Random	3.56(2.51,4.62)		81.44		5.39	0.00
MSQ-28item	3	901	Random	5.38(4.20,6.57)		90.80		28.21	0.00
MSQ-R	4	1,310	Random	5.46(5.17,5.74)		0.00		0.05	0.00
MSQR-CV	32	12,050	Random	5.22(5.01,5.43)		14.29		25.17	0.00
Department					0.31				
Emergency	2	345	Random	3.26(2.01,4.51)		6.11		1.07	0.00
Hemodialysis	1	123	Random	4.88(3.76,6.00)		-		-	0.00
Hospice	2	414	Random	4.86(3.81,5.91)		0.00		0.03	0.00
ICU	19	3,670	Random	4.18(3.73,4.62)		58.11		37.70	0.00
Mixed	61	19,831	Random	4.54(4.27,4.81)		89.55		555.85	0.00
Neurosurgery	1	197	Random	4.67(2.73,6.61)		-		-	0.00
Operating room	2	564	Random	5.24(4.07,6.41)		0.00		0.09	0.00
Pediatric	4	1,290	Random	4.55(3.66,5.45)		23.79		3.80	0.00
Psychiatry	5	1,578	Random	4.19(3.26,5.12)		63.66		11.54	0.00
Oncology	4	1,276	Random	5.03(2.73,6.61)		44.83		5.43	0.00
Geriatrics	1	99	Random	5.24(4.18,6.30)		-		-	0.00
Gender					0.84				
Female	31	8,246	Random	4.11(3.76,4.47)		76.14		152.21	0.00
Male	30	2,146	Random	4.07(3.74,4.39)		75.23		180.26	0.00
Educational level					0.14				
Bachelor or lower	18	7,359	Random	4.08(3.78,4.37)		86.20		835.90	0.00
Master or above	21	837	Random	4.44(4.05,4.83)		81.50		63.13	0.00
Work experience					0.07				
≤ 5 years	13	1,939	Random	3.78(3.32,4.23)		75.01		54.21	0.00
6–10 years	8	836	Random	4.51(3.80,5.23)		63.76		28.05	0.00
≥ 10 years	19	2,927	Random	4.40(4.03,4.78)		70.35		82.95	0.00
Learning experiences in MS					0.56				
Yes	11	2,556	Random	4.65(4.21,5.10)		35.76		19.21	0.00
No	11	1,287	Random	4.48(4.09,4.87)		0.00		11.07	0.00

Note: *P*^*a*^ value for the between-subgroup difference, *P*^*b*^ value for the heterogeneity within subgroups by Q test

### Sensitivity analysis

**Fig. 3:**
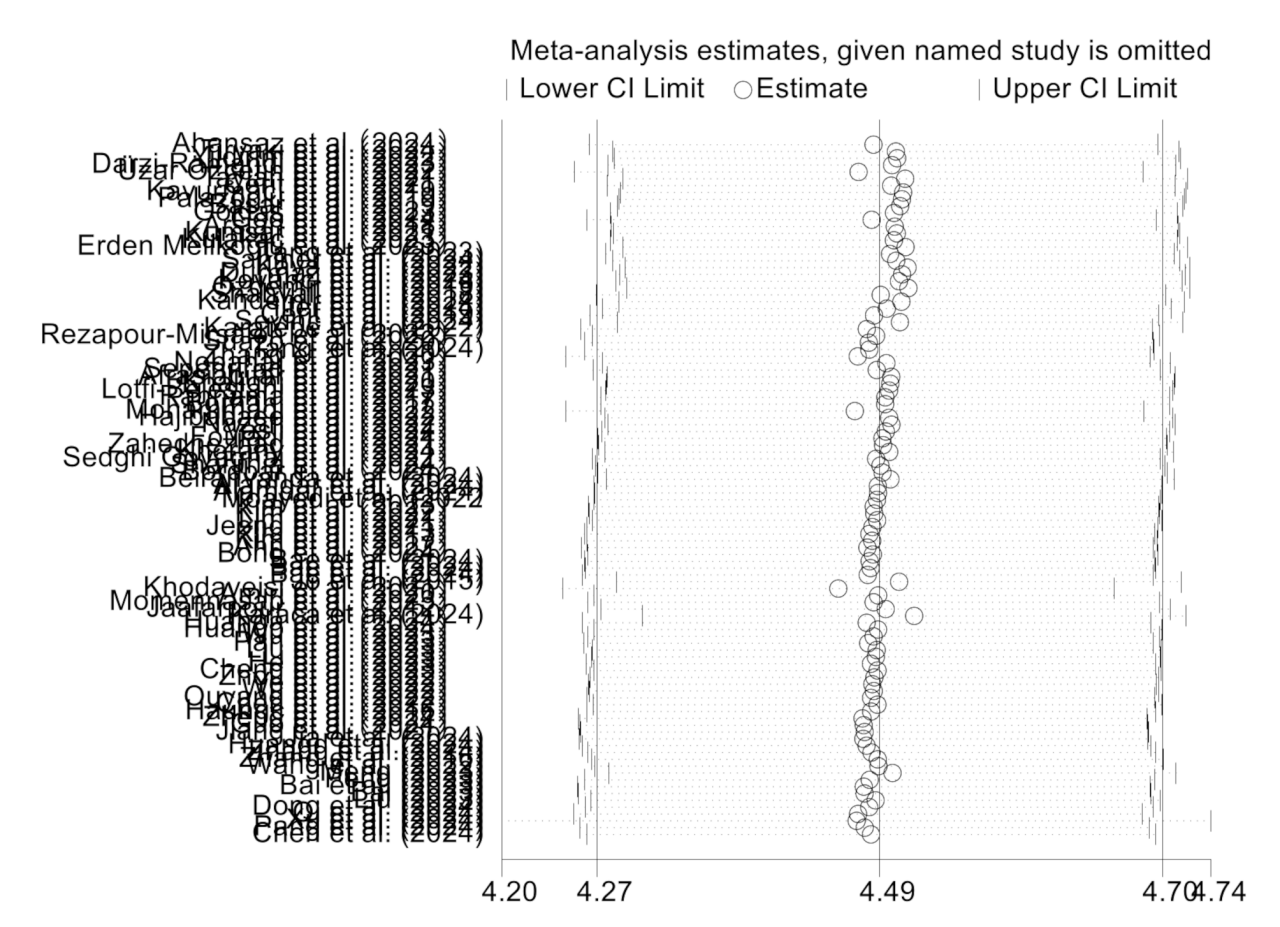
The results of sensitivity analysis

A sensitivity analysis was conducted by sequentially omitting each study and recalculating the pooled mean score to assess the robustness of the findings. No significant changes were observed, indicating the stability of the findings (Fig. 3).

### Publication bias

**Fig. 4:**
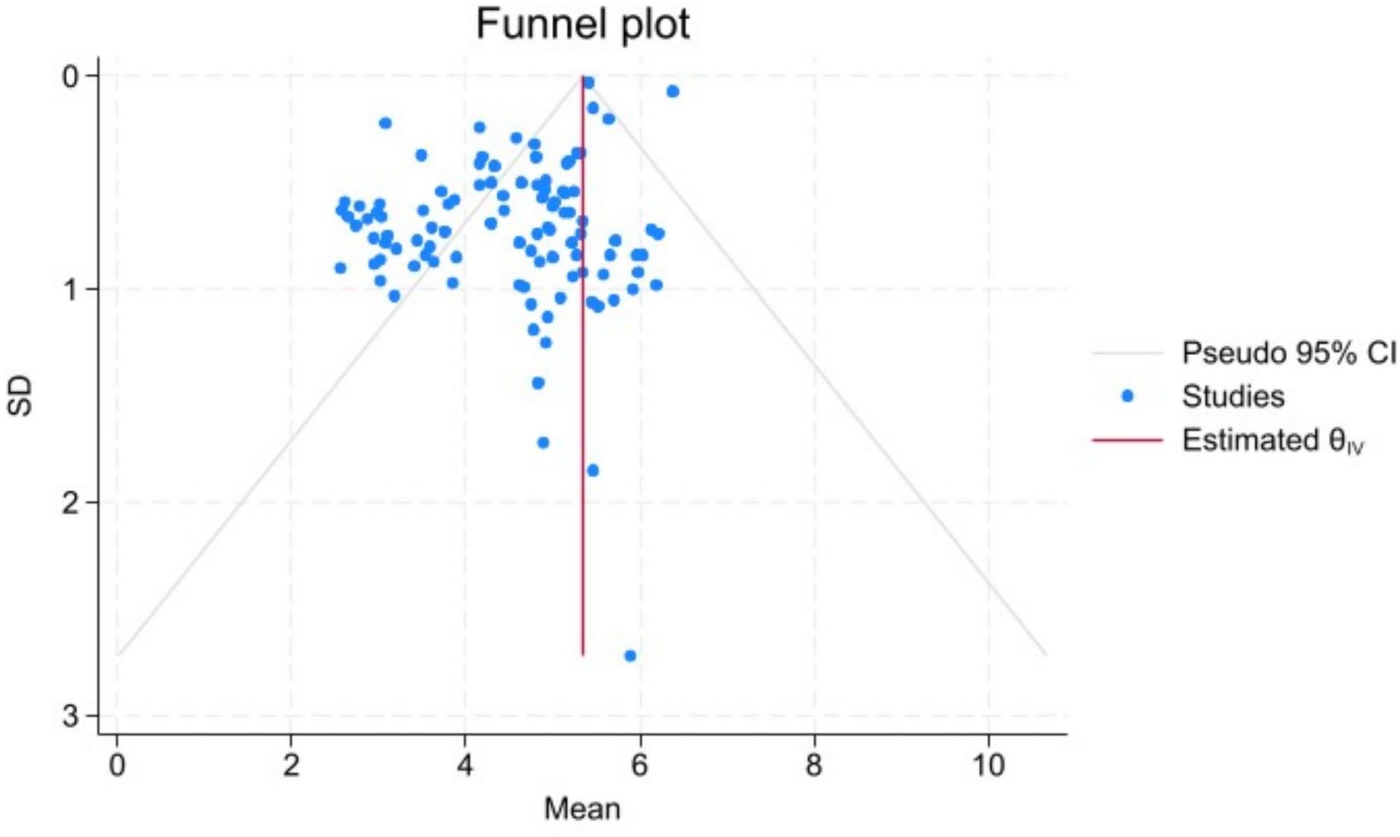
The Funnel Plot

**Fig. 5:**
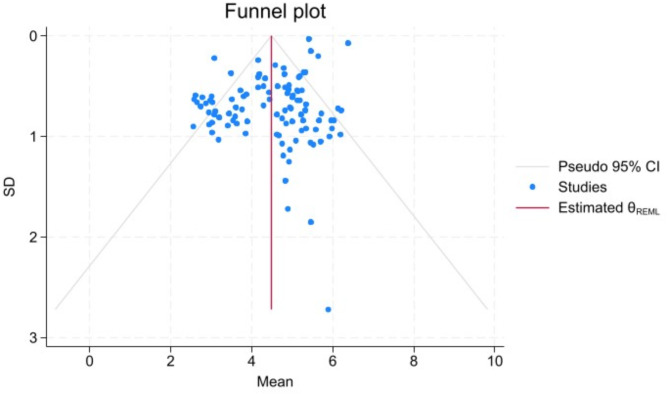
The cut-and-fill method for correcting the moral - sensitivity level of nurses (Right)

**Fig. 6:**
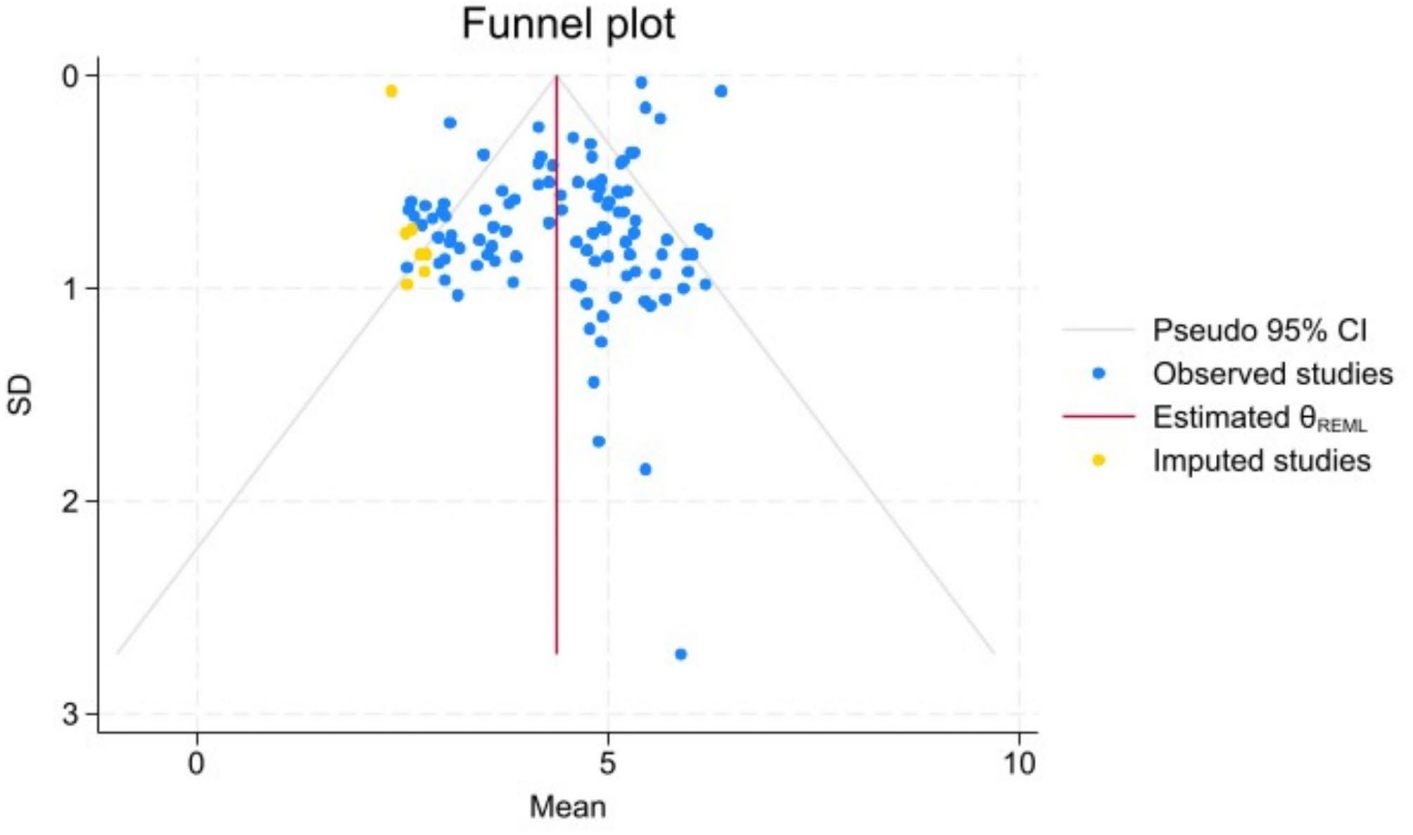
The cut-and-fill method for correcting the moral - sensitivity level of nurses (Left)

Egger’s test yielded a statistically significant *P*-value (*P* = 0.001), indicating potential publication bias. To address this, the cut-and-fill method was applied for correction. After adjusting for bias by filling in both the left and right sides, the pooled mean score of nurses for MS were 4.36 [95% *CI* (4.16, 4.58)] and 4.49 [95% *CI* (4.29, 4.70)] respectively. This indicates that publication bias did not undermine the reliability of our results (Figs. 4, 5 and 6).

## Discussion

This review included 99 cross-sectional studies [13, 20, 24–27, 29, 31, 42–132], all of which were evaluated using the JBI Critical Appraisal Tool. The quality of included studies ranged from moderate to high. All studies clearly defined their subjects, inclusion criteria, measurement methods, statistical analyses, and outcomes. However, 61 studies [13, 20, 24, 26, 27, 29, 31, 44–48, 52, 54, 57, 58, 60–62, 64–67, 69, 72–74, 77, 80–83, 85, 87–91, 93–98, 100, 101, 103, 104, 106, 109, 110, 112, 114, 120, 122–124, 127–129, 131] were rated as moderate quality due to limitations in their research design. Specifically, these studies failed to adequately account for potential confounding factors in their measurements and analyses, which might have influenced their results. The absence of proper control for these confounders could lead to over- or underestimation of the relationships between the key variables, thereby diminishing the reliability of the findings. To enhance the quality of future research, it is crucial to address these confounding factors properly.

To the best of our knowledge, this is the first meta-analysis to assess MS levels among nurses. The results of this review indicated that the level of MS among nurses was moderate. However, considerable heterogeneity was observed in this meta-analysis. To further explore the sources of heterogeneity, meta-regression and subgroup analyses were performed, considering factors such as country, department, assessment instrument, gender, education level, work experience, learning experience in MS, publication year, and sample size. Furthermore, sensitivity analysis was conducted to assess the robustness of the results.

Our results demonstrated that the pooled mean score for MS among nurses was 4.49 [95% *CI* (4.29, 4.70)], which represents a moderate level and is consistent with the results of previous studies [43, 53, 85, 133]. This finding suggests that nurses can identify moral issues and reduce ethical distress. However, the clinical scenarios faced by nurses are much more complex than we might imagine, and there remains room to improve the MS level. As an important form of moral awareness, MS plays a crucial role in promoting ethical conduct among nurses. Nursing managers and educators must prioritize the enhancement of MS levels among nurses.

Meta-regression results demonstrated that the assessment instrument, country, and department were statistically significant. This finding suggests that substantial variation in assessment instrument across studies may be a significant contributor to the observed heterogeneity. Another potential source of heterogeneity could be the fact that the studies involved nurses from different countries, each with unique cultural, ethnic, social, and economic contexts. Furthermore, the differences in departments where nurses are employed may also serve as an important source of heterogeneity, given the variations in work intensity and organizational climate across departments. Although meta-regression analyses were conducted to explore the sources of heterogeneity, the limited number of included studies may have reduced the statistical power of the meta-regression in identifying these sources.

Subgroup analysis revealed significant differences in the pooled mean scores of nurses’ MS across countries, and assessment instrument. At the first place, subgroup analysis revealed notable differences between countries, which may be strongly linked to cultural variation. While nursing practices share commonalities worldwide, the MS level among nurses varies and is influenced by distinct cultural contexts in each country [134]. Additionally, institutional policies and societal expectations further contribute to these variations, resulting in differing MS levels across nations [135]. Lastly, the meta-analysis included studies that used five different MS assessment instruments. The pooled mean score reported using the various assessment instruments varied widely, ranging from 5.46 (the MSQ-R scale) to 3.57 (the MSQ scale). All included studies assessed the level of MS among nurses using the MSQ scale or its adapted versions, which differed in the number of items compared to the original version following its translation and adaptation. This may have contributed to the discrepancies observed [136]. Furthermore, objective assessment of MS level remains limited, as it primarily relies on individual reports and self-reported rating scales, which may exacerbate these discrepancies [14].

Although the results of the meta-subgroup analysis indicated no statistically significant differences in terms of country, gender, education level, work experience and whether nurses had undergone MS training, we still consider it necessary to further discuss these factors. First, the level of MS among nurses varied significantly depending on the department in which they worked. The meta-analysis revealed that the operating room nurses exhibited the pooled mean score of 5.24 [95% CI (4.07, 6.41)], while ICU nurses presented the pooled mean score of 3.26 [95% *CI* (2.01, 4.51)], which indicates a relatively high level and moderate-low level of MS among nurses. ICU nurses often experience high work demands and long working hours, leading to burnout and fatigue, which may diminish their MS levels [137, 138]. It is well-documented that the emergency department is characterized by high pressure, frequent demands, and rapid decision-making, all of which contribute to a significant prevalence of nurse burnout and a heightened incidence of moral distress, thereby weakening the MS level [139–141]. Therefore, to improve MS levels, the strategic allocation of human resources and the provision of emotional and psychological support for nurses should be emphasized, as this will help address both the ethical and emotional challenges faced by nurses in high-pressure environments, such as the emergency and ICU departments. Secondly, the pooled mean score for females was 4.11[95% *CI* (3.76, 4.77)], and for males, it was 4.07 [95% *CI* (3.74, 4.39)], without significant differences. However, previous studies have revealed differences between genders at the MS level [75, 134, 142]. The following factors may account for the observed differences in MS between males and females across various studies: MS was positively correlated with empathy, and female nurses, who possess more delicate emotions, a stronger capacity for empathy, and greater sensitivity to moral issues, tend to exhibit higher MS level [143, 144]. Nonetheless, existing literature indicates that females are more susceptible to moral distress, which can result in empathy fatigue and adversely affect their MS levels [17, 145]. We contend that it is essential to develop and implement ethical training programs considering gender differences. Thirdly, our results depicted that nurses with a bachelor’s degree or lower had a pooled mean score of 4.08 [95% *CI* (3.78, 4.37)], while those with a master’s degree or higher had a pooled mean score of 4.44 [95% *CI* (4.05, 4.83)]. Previous studies have indicated that individuals with a master’s degree generally possess better critical thinking skills and enhanced ability to manage clinical problems, which subsequently leads to a higher MS level and less moral distress [146–149]. Accordingly, we suggest strengthening MS training for nurses with lower academic qualifications. Fourthly, the results revealed that the pooled mean scores of nurses with work experience of ≤ 5 years, 6–10 years, and ≥ 10 years were 3.78 [95% *CI* (3.32, 4.23)], 4.51 [95% *CI* (3.80, 5.23)], and 4.40 [95% *CI* (4.03, 4.78)], respectively. With the accumulation of clinical experience, nurses become more experienced in caring for patients and are more likely to recognize and address moral issues. This is consistent with the findings of Yildirim et al. [54] and Hognestadet et al. [150], who revealed that the richer the clinical experience of nurses, the higher their MS. However, Arslan et al. [78] stated that, as the number of working years increased, moral sensitivities decreased in the holistic approach subscale. This is related to compassion fatigue. Peters et al. [151] posited that, with an increase in the number of working years, the incidence of compassion fatigue increases, which exerts a reverse impact on MS. Consequently, we recommend that nursing managers develop training programs at different levels to meet the needs of nurses with varying seniority levels. Finally, the results indicated that nurses who underwent moral training exhibited a pooled mean score of 4.65 [95% *CI* (4.21, 5.10)], compared to a pooled mean score of 4.48 [95% *CI* (4.09, 4.87)] for those who did not participate in moral training. Multiple studies have demonstrated that nurses who participated in moral training scored higher on MS than those who did not [43, 55, 152]. Besides, several studies have confirmed that moral training effectively improves the MS level and serves as a robust strategy for alleviating moral distress [60, 153–155]. Nurses with moral training can identify and resolve ethical issues effectively. Consequently, we advocate that nurses continue to learn about and strengthen their moral education.

Overall, our review found that the level of MS among nurses was moderate, indicating the need for further improvement. The results from current studies clearly depict that MS is influenced by various factors. Based on this, the following recommendations can be made to enhance MS. First, given cultural differences between countries, it is essential to implement ethics training programs tailored to the specific cultural context of each nation. Second, it is of paramount importance for nursing administrators to prioritize ethics training in departments with relatively low MS levels, such as ICU and emergency departments. Third, nursing educators and administrators should design and deliver personalized ethics training that considers the individual characteristics of nurses, including gender and years of experience, to mitigate moral dilemmas and enhance MS. Finally, both the accumulation of clinical experience and higher education levels were found to positively influence MS. As such, we advocate for a commitment to lifelong learning, with nurses actively engaging in ongoing moral education throughout their professional careers.

### Strengths and limitations

This review has several advantages. To our knowledge, this is the first study to assess present MS levels among nurses by conducting a quantitative meta-analysis. We used the JBI’s Critical Appraisal Tool to assess the methodological quality of all the included studies, and the results were moderate to high.

However, this study has several limitations. First, because all included studies were cross-sectional, it was difficult to control for corresponding confounding factors. For instance, factors such as individual traits (such as gender) and cultural background (such as country) can significantly influence MS. Based on these factors, the heterogeneity in our meta-analysis was relatively high. Second, discrepancies in the number of items and dimensions across the assessment instrument made it infeasible to aggregate the total or dimension-specific scores. The variation in the structure of the instruments hindered a unified approach to data synthesis, which could have otherwise allowed for a more comprehensive evaluation. Third, all included studies were published in Chinese or English, which means that relevant research published in other languages may have been overlooked, potentially introducing language bias. This limitation may have resulted in the exclusion of important findings or perspectives that may have been captured in studies published in other languages. Fourth, the grey literature databases had not been searched, which might have led to publication bias. Therefore, our findings may be limited in scope and should be cautiously interpreted.

Notwithstanding the limitations of our review, we endeavored to ensure that our approach remains methodical and exhaustive. By systematically synthesizing pertinent findings, we aimed to provide practical, evidence-based guidance that can inform the future evolution and refinement of moral training programs for healthcare professionals. Moreover, we hope to provide critical insights into areas that require further investigation and enhancement, thereby contributing to the advancement of MS and the promotion of ethical practices within the nursing profession.

## Conclusion

In summary, our review aimed to systematically assess MS levels. The results indicated that, while nurses generally demonstrate moderate MS, there is potential for further improvement. As the largest group of healthcare service providers, nurses need to exhibit an adequate MS level that supports their role in providing quality care. Accordingly, implementing moral training programs could help to enhance MS among nurses. When designing and implementing such programs, it is essential to consider factors such as national cultural differences, departmental variations, and individual characteristics, including gender and educational experience, to ensure focused and effective improvement of MS among nurses.

## Electronic supplementary material

Below is the link to the electronic supplementary material.

Supplementary Material 1

## Data Availability

All data generated or analysed during this study are included in this published article and its supplementary information files.

## Abbreviations

MS: Moral sensitivity
CNKI: China Knowledge Resource Integrated Database
VIP: Weipu Database
CBM: Chinese Biomedical Database
CMCC: Chinese Medical Journal Full Text Database
MSQ: The moral sensitivity questionnaire
JBI: Joanna Briggs Institute
Cis: Confidence intervals
MSQ-R: The moral sensitivity questionnaire-revised version
L-MSQ: Lützén moral sensitivity questionnaire
K-MSQ: The Korean version of the moral sensitivity questionnaire
MSQR-CV: The Chinese moral sensitivity questionnaire-revised version
ICU: Intensive care unit
