# Supplementary material for: The level of moral sensitivity among nurses: a systematic review and meta-analysis
Source: BMC Nurs. 2025 Mar 25;24:321. doi: 10.1186/s12912-025-02892-6 (PMC11938698; doi:10.1186/s12912-025-02892-6)
Supplement: Supplementary file 1 — Supplementary Material 1 [file 12912_2025_2892_MOESM1_ESM.docx]

**Appendix A: Search strategy**

| **Database** | **Search terms** | **Results** |
| --- | --- | --- |
| PubMed | #1 Nurses[MeSH Terms]  #2 nurs*[Title/Abstract]  #3 #1 OR #2  #4 ((moral[Title/Abstract]) OR (Ethic*[Title/Abstract])) AND (sensit*[Title/Abstract])  #5 #3 AND #4 | 820 |
| Cochrane | #1 (nurs*):ti,ab,kw  #2 ((moral OR ethic) AND sensitivity):ti,ab,kw  #3 #1 AND #2 | 123 |
| Embase | #1 Nurses/de  #2 nurs*:ab,ti  #3 #1 OR #2  #4 ((moral):ab,ti OR (ethic):ab,ti) AND (sensitivity):ab,ti  #5 #3 AND #4 | 429 |
| Web of Science | #1 TS=Nurs*  #2 TS=((moral or ethic*) AND sensitivity)  #3 #1 AND #2 | 416 |
| CINAHL | #1 AB=Nurs*  #2 AB=((moral or ethic*) AND sensitivity)  #3 #1 AND #2 | 577 |
| Scopus | #1 TITLE-ABS-KEY (moral or ethic*)  #2 TITLE-ABS-KEY( sensitivity)  #3 #1 AND #2  #4 TITLE-ABS-KEY (Nurs*)  #5 #3 AND #4 | 746 |
| Medline | #1 nurs*.ab.  #2 ((moral or ethic*) AND sensitivity).ab.  #3 #1 AND #2 | 419 |
| CNKI | #1 SU=('护士'+'护理'+'临床护士'+'护理人员')  #2 SU=('道德敏感性'+'伦理敏感性')  #3 #1 AND #2 | 60 |
| Wanfang | #1 (主题:("护理") or 主题:("临床护士") or 主题:("护士") or 主题:("护理人员"))  #2 (主题:("道德敏感性") or 主题:("伦理敏感性"))  #3 #1 AND #2 | 55 |
| VIP | #1 M=(护士 OR 护理 OR 临床护士 OR 护理人员)  #2 M=(道德敏感性 OR 伦理敏感性)  #3 #1AND #2 | 37 |
| CBM | #1 ("护士"[常用字段:智能] OR "护理"[常用字段:智能] OR "护理人员"[常用字段:智能] OR "临床护士"[常用字段:智能])  #2 ( "道德敏感性"[常用字段:智能] OR "伦理敏感性"[常用字段:智能])  #3 #1 AND #2 | 50 |
| CMCC | #1 (主题=("护士" OR "护理" OR "护理人员" OR "临床护士"))  #2 (主题=("道德敏感性" OR "伦理敏感性"))  #3 #1 AND #2 | 2 |

**Appendix B.** **The results of quality assessment**

| **Study** | **JBI critical appraisal checklist for analytical cross-section studies** | | | | | | | | **Result** |
| --- | --- | --- | --- | --- | --- | --- | --- | --- | --- |
|  | C1 | C2 | C3 | C4 | C5 | C6 | C7 | C8 |  |
| Ahansaz et al. (2024) | Y | Y | NA | Y | N | N | Y | Y | Moderate quality |
| Tiryaki et al. (2024) | Y | Y | NA | Y | Y | Y | Y | Y | High quality |
| Yildirim et al. (2022) | Y | Y | NA | Y | N | N | Y | Y | Moderate quality |
| Darzi-Ramandi et al. (2023) | Y | Y | NA | Y | Y | Y | Y | Y | High quality |
| Üzar Özçetin et al. (2022) | Y | Y | NA | Y | N | N | Y | Y | Moderate quality |
| Taylan et al. (2021) | Y | Y | NA | Y | N | N | Y | Y | Moderate quality |
| Cerit et al. (2021) | Y | Y | NA | Y | N | N | Y | Y | Moderate quality |
| Kavurmacı et al. (2019) | Y | Y | NA | Y | Y | Y | Y | Y | High quality |
| Palazoglu et al. (2019) | Y | Y | NA | Y | Y | Y | Y | Y | High quality |
| Basar et al. (2019) | Y | Y | NA | Y | Y | Y | Y | Y | High quality |
| Goktas et al. (2023) | Y | Y | NA | Y | Y | Y | Y | Y | High quality |
| Goo et al. (2024) | Y | Y | NA | Y | N | N | Y | Y | Moderate quality |
| Arslan et al. (2018) | Y | Y | NA | Y | Y | Y | Y | Y | High quality |
| Kumsar et al. (2021) | Y | Y | NA | Y | Y | Y | Y | Y | High quality |
| Kulakaç et al. (2023) | Y | Y | NA | Y | N | N | Y | Y | Moderate quality |
| Erden Melikoğlu et al. (2023) | Y | Y | NA | Y | N | N | Y | Y | Moderate quality |
| Tang et al. (2023) | Y | Y | NA | Y | Y | Y | Y | Y | High quality |
| Sahiner et al. (2024) | Y | Y | NA | Y | Y | Y | Y | Y | High quality |
| Kaya et al. (2022) | Y | Y | NA | Y | Y | Y | Y | Y | High quality |
| Durmaz et al. (2023) | Y | Y | NA | Y | N | N | Y | Y | Moderate quality |
| Kovanci et al. (2024) | Y | Y | NA | Y | N | N | Y | Y | Moderate quality |
| Ozdemir et al. (2019) | Y | Y | NA | Y | Y | Y | Y | Y | High quality |
| Shahvali et al. (2018) | Y | Y | NA | Y | N | N | Y | Y | Moderate quality |
| Kandemir et al. (2024) | Y | Y | NA | Y | N | N | Y | Y | Moderate quality |
| Ilter et al. (2024) | Y | Y | NA | Y | Y | Y | Y | Y | High quality |
| Cerit et al. (2019) | Y | Y | NA | Y | N | N | Y | Y | Moderate quality |
| Sevinç et al. (2024) | Y | Y | NA | Y | N | N | Y | Y | Moderate quality |
| Karatepe et al. (2022) | Y | Y | NA | Y | N | N | Y | Y | Moderate quality |
| Rezapour-Mirsaleh et al. (2022) | Y | Y | NA | Y | N | N | Y | Y | Moderate quality |
| Suazo et al. (2020) | Y | Y | NA | Y | N | N | Y | Y | Moderate quality |
| Tang et al. (2024） | Y | Y | NA | Y | Y | Y | Y | Y | High quality |
| Nobahar et al. (2023) | Y | Y | NA | Y | N | N | Y | Y | Moderate quality |
| Sepehrirad et al. (2021) | Y | Y | NA | Y | N | N | Y | Y | Moderate quality |
| Afrasiabifar et al. (2021) | Y | Y | NA | Y | N | N | Y | Y | Moderate quality |
| Khalighi et al. (2020) | Y | Y | NA | Y | Y | Y | Y | Y | High quality |
| Lotfi-Bejestani et al. (2023) | Y | Y | NA | Y | N | N | Y | Y | Moderate quality |
| Rahnama et al. (2017) | Y | Y | NA | Y | N | N | Y | Y | Moderate quality |
| Borhani et al. (2015) | Y | Y | NA | Y | N | N | Y | Y | Moderate quality |
| Mohammadi et al. (2022) | Y | Y | NA | Y | Y | Y | Y | Y | High quality |
| Hajibabaee et al. (2022) | Y | Y | NA | Y | N | N | Y | Y | Moderate quality |
| Nazari et al. (2022) | Y | Y | NA | Y | Y | Y | Y | Y | High quality |
| Vasli et al. (2024) | Y | Y | NA | Y | N | N | Y | Y | Moderate quality |
| Fouladi et al. (2024) | Y | Y | NA | Y | N | N | Y | Y | Moderate quality |
| Zahednezhad et al. (2021) | Y | Y | NA | Y | N | N | Y | Y | Moderate quality |
| Khorany et al. (2024) | Y | Y | NA | Y | N | N | Y | Y | Moderate quality |
| Sedghi Goyaghaj et al. (2022) | Y | Y | NA | Y | N | N | Y | Y | Moderate quality |
| Sharifnia et al. (2024) | Y | Y | NA | Y | Y | Y | Y | Y | High quality |
| Bordbar et al. (2024) | Y | Y | NA | Y | Y | Y | Y | Y | High quality |
| Beiranvanda et al. (2024) | Y | Y | NA | Y | Y | Y | Y | Y | High quality |
| Alamdar et al. (2024) | Y | Y | NA | Y | Y | Y | Y | Y | High quality |
| Moayedi et al. (2022) | Y | Y | NA | Y | Y | Y | Y | Y | High quality |
| Kim et al. (2023) | Y | Y | NA | Y | N | N | Y | Y | Moderate quality |
| Kim et al. (2022) | Y | Y | NA | Y | N | N | Y | Y | Moderate quality |
| Lim et al. (2021) | Y | Y | NA | Y | Y | Y | Y | Y | High quality |
| Jeong et al. (2021) | Y | Y | NA | Y | N | N | Y | Y | Moderate quality |
| Kim et al. (2013) | Y | Y | NA | Y | N | N | Y | Y | Moderate quality |
| Kim et al. (2017) | Y | Y | NA | Y | N | N | Y | Y | Moderate quality |
| Ahn et al. (2022) | Y | Y | NA | Y | N | N | Y | Y | Moderate quality |
| Bong et al. (2024) | Y | Y | NA | Y | N | N | Y | Y | Moderate quality |
| Bae et al. (2024) | Y | Y | NA | Y | N | N | Y | Y | Moderate quality |
| Jo et al. (2015) | Y | Y | NA | Y | N | N | Y | Y | Moderate quality |
| Khodaveisi et al. (2021) | Y | Y | NA | Y | N | N | Y | Y | Moderate quality |
| Amiri et al. (2020) | Y | Y | NA | Y | N | N | Y | Y | Moderate quality |
| Momennasab et al. (2023) | Y | Y | NA | Y | N | N | Y | Y | Moderate quality |
| Jaafarpour et al. (2012) | Y | Y | NA | Y | N | N | Y | Y | Moderate quality |
| Karaca et al. (2024) | Y | Y | NA | Y | Y | Y | Y | Y | High quality |
| Zhang et al. (2020) | Y | Y | NA | Y | N | N | Y | Y | Moderate quality |
| Huang^a^ et al. (2024) | Y | Y | NA | Y | N | N | Y | Y | Moderate quality |
| Huang^b^ et al. (2024) | Y | Y | NA | Y | Y | Y | Y | Y | High quality |
| Wu et al. (2023) | Y | Y | NA | Y | N | N | Y | Y | Moderate quality |
| Pan et al. (2023) | Y | Y | NA | Y | N | N | Y | Y | Moderate quality |
| Liu et al. (2023) | Y | Y | NA | Y | N | N | Y | Y | Moderate quality |
| Li et al. (2023) | Y | Y | NA | Y | Y | Y | Y | Y | High quality |
| He et al. (2023) | Y | Y | NA | Y | Y | Y | Y | Y | High quality |
| Cheng et al. (2023) | Y | Y | NA | Y | N | N | Y | Y | Moderate quality |
| Zhou et al. (2022) | Y | Y | NA | Y | Y | Y | Y | Y | High quality |
| Ye et al. (2022) | Y | Y | NA | Y | Y | Y | Y | Y | High quality |
| Wu et al. (2022) | Y | Y | NA | Y | Y | Y | Y | Y | High quality |
| Ouyang et al. (2022) | Y | Y | NA | Y | Y | Y | Y | Y | High quality |
| Chen et al. (2019) | Y | Y | NA | Y | N | N | Y | Y | Moderate quality |
| Huang^c^ et al. (2016) | Y | Y | NA | Y | N | N | Y | Y | Moderate quality |
| Zheng et al. (2024) | Y | Y | NA | Y | N | N | Y | Y | Moderate quality |
| Guo et al. (2024) | Y | Y | NA | Y | N | N | Y | Y | Moderate quality |
| Jiang et al. (2021) | Y | Y | NA | Y | N | N | Y | Y | Moderate quality |
| Jia et al. (2024) | Y | Y | NA | Y | Y | Y | Y | Y | High quality |
| Huang^d^ et al. (2024) | Y | Y | NA | Y | Y | Y | Y | Y | High quality |
| Zhang et al.(2024) | Y | Y | NA | Y | N | N | Y | Y | Moderate quality |
| Zhang et al. (2016) | Y | Y | NA | Y | N | N | Y | Y | Moderate quality |
| Wang et al. (2022) | Y | Y | NA | Y | N | N | Y | Y | Moderate quality |
| Meng (2023) | Y | Y | NA | Y | N | N | Y | Y | Moderate quality |
| Peng (2023) | Y | Y | NA | Y | Y | Y | Y | Y | High quality |
| Bai et al. (2023) | Y | Y | NA | Y | Y | Y | Y | Y | High quality |
| Bai (2023) | Y | Y | NA | Y | Y | Y | Y | Y | High quality |
| Liu (2023) | Y | Y | NA | Y | N | N | Y | Y | Moderate quality |
| Dong et al. (2024) | Y | Y | NA | Y | N | N | Y | Y | Moderate quality |
| Qi et al. (2024) | Y | Y | NA | Y | N | N | Y | Y | Moderate quality |
| Xu et al. (2024) | Y | Y | NA | Y | N | N | Y | Y | Moderate quality |
| Pang et al. (2024) | Y | Y | NA | Y | Y | Y | Y | Y | High quality |
| Chen et al. (2024) | Y | Y | NA | Y | Y | Y | Y | Y | High quality |

Note: Y: Yes; N: No; UC: Unclear; NA: Not applicable; C1: Were the criteria for inclusion in the sample clearly defined?; C2: Were the study subjects and the setting described in detail?; C3: Was the exposure measured in a valid and reliable way?; C4: Were objective, standard criteria used for measurement of the condition?; C5: Were confounding factors identified?; C6: Were strategies to deal with confounding factors stated?; C7:Were the outcomes measured in a valid and reliable way?; C8: Was appropriate statistical analysis used?
